# Reproducibility and transparency: why following MISEV guidelines is beneficial for the studies on EVs and brain barriers

**DOI:** 10.20517/evcna.2024.63

**Published:** 2025-07-03

**Authors:** Julien Saint-Pol

**Affiliations:** Université d’Artois, Blood-Brain Barrier laboratory (LBHE - UR2465), Faculté des Sciences Jean Perrin, F-62300 Lens, France.

**Keywords:** Extracellular vesicles, MISEV, brain barriers, blood-brain barrier, blood-CSF barrier, blood-retina barrier, arachnoid-CSF barrier, blood-nerve barrier

Over the past two decades, research on extracellular vesicles (EVs) has surged, driven by their role as key regulators of intercellular communication, pathological biomarkers, and their potential use as therapeutic vectors. A particular area of interest is their ability to traverse biological barriers, notably the brain barriers, such as the blood-brain barrier (BBB), to optimize the delivery of pharmacological and epigenetic regulatory compounds to the brain. However, to ensure the reproducibility and transparency of findings and to standardize EV nomenclature, it is imperative to adopt rigorous research methodologies. Under the guidance of the International Society of Extracellular Vesicles (ISEV), the Minimum Information for Studies of Extracellular Vesicles (MISEV) was first published in 2014 and updated in 2018 and 2023 to reflect advances in technology and theory. The objective of this editorial is to inform researchers and journal reviewers of the key considerations necessary for producing high-quality publications in the EV and brain barrier research domains.

## THE BRAIN BARRIERS AS A PROVIDENTIAL PLAYGROUND FOR EV STUDIES

**Figure 1:**
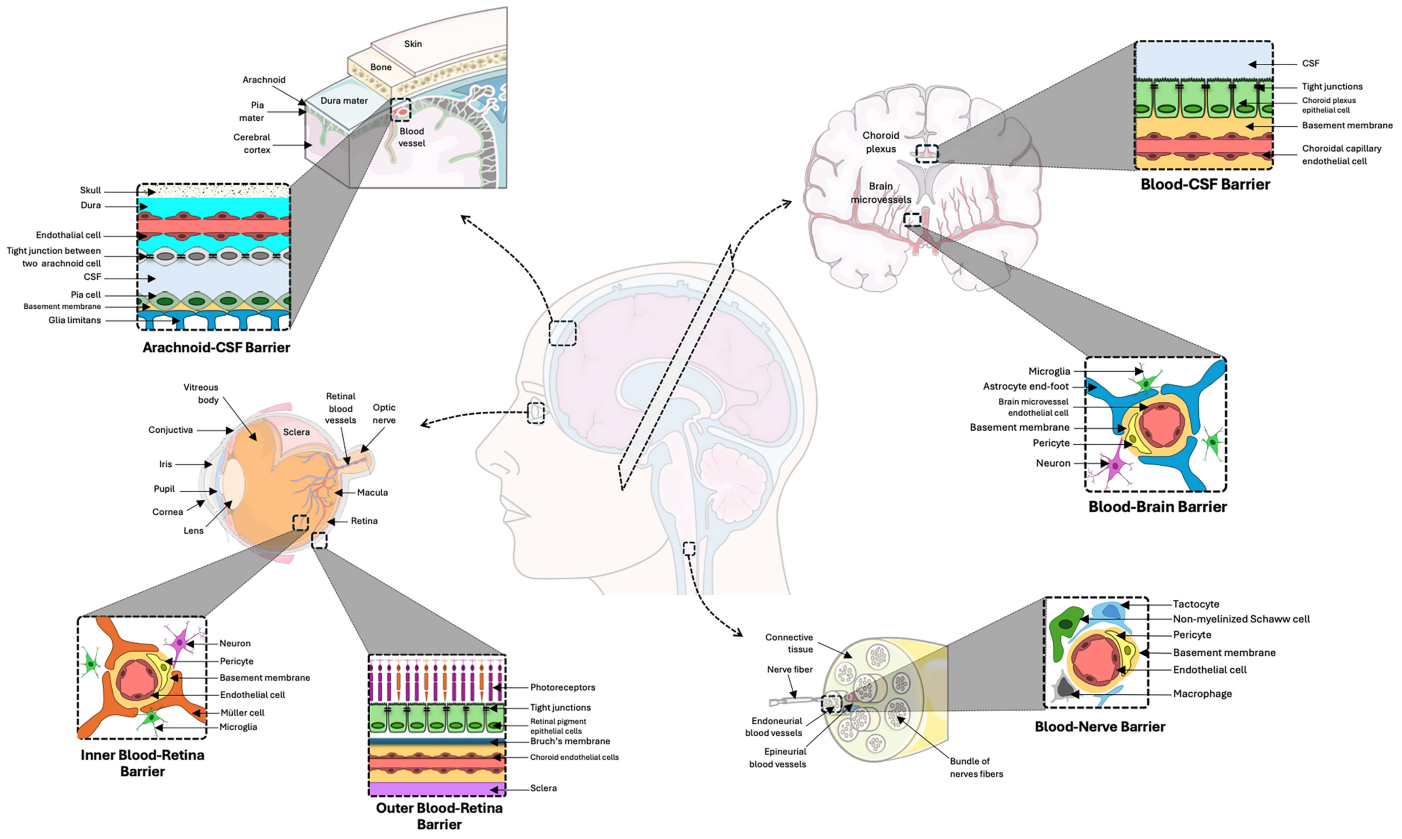
Location and main features of the brain barriers. Brain barriers serve as protective interfaces for both the central and peripheral nervous systems. While their anatomical locations vary, they share structural similarities and undergo comparable postnatal maturation processes. These features collectively contribute to their essential role in safeguarding the nervous system and maintaining its homeostasis. For further details, see reviews^[1-6]^.

The central and peripheral nervous systems are protected by five specialized barriers - the BBB, blood-cerebrospinal fluid barrier (BCSFB), arachnoid-cerebrospinal fluid (CFS) barrier, blood-retina barrier (BRB), and blood-nerve barrier (BNB) - that regulate nutrient transport, waste clearance, and homeostasis^[1-6]^ [Figure 1]. While structurally distinct, these barriers share a dependence on intercellular communication to maintain their integrity.

The BBB, the most studied, is composed of endothelial cells sealed by tight junctions, supported by astrocytes, pericytes, and neurons forming the neurovascular unit (NVU). It restricts paracellular transport and employs efflux pumps (e.g., P-gp, BCRP) and enzymes (e.g., MAO, CYPs) to protect the brain; dysfunction is linked to neurodegenerative diseases^[7-11]^. The BCSFB, formed by choroid plexus epithelial cells, fenestrated endothelial cells, and immune components, regulates CSF production and molecular exchange^[2,5,12]^. The arachnoid-CSF barrier, located within the meninges, protects CSF from toxic infiltration^[1]^. The BRB consists of inner endothelial and outer retinal pigment epithelial cells (RPECs), reinforced by Müller cells and pericytes, and employs transporters such as GLUT1 and P-gp^[4,13]^. The BNB comprises endoneurial endothelial cells, supported by Schwann cells, tactocytes, and perineurial cells, to regulate the peripheral nerve microenvironment^[14,15]^.

Numerous experimental models have been developed to study these barriers. *In vitro* systems, including co-cultures and organ-on-chip platforms, are widely used but often lack full physiological complexity^[16-18]^. Barrier-specific *in vitro* models, e.g., BBB co- or tri-cultures, retinal pigment epithelial cells (RPECs) monolayers for the BRB, and choroid plexus cultures for the BCSFB, enable targeted studies, though glial components are often underrepresented^[2,4,6,19-21]^. In vivo rodent models remain essential for understanding barrier dynamics and drug delivery but face translational limitations due to interspecies differences^[22-25]^. The zebrafish has emerged as a complementary model, offering optical transparency, conserved barrier features, and suitability for developmental and pharmacological studies^[26-29]^. Meanwhile, human stem cell-derived models and 3D brain/retina organoids are improving *in vitro* relevance by better replicating human transporter expression and cellular architecture, although challenges in reproducibility and scalability persist^[30-32]^.

In parallel, EVs have gained attention as key players in brain barriers research. These lipid bilayer-enclosed vesicles, secreted by nearly all eukaryotic and prokaryotic cells, include exosomes and ectosomes and are found in fluids such as blood, CSF, urine, and saliva^[33,34]^. EVs carry bioactive cargo - proteins, lipids, mRNAs, miRNAs, lncRNAs, and DNA fragments - and mediate intercellular communication through membrane fusion, endocytosis, or receptor binding^[35-37]^. Owing to their presence in accessible fluids and tissue-specific molecular signatures, EVs are promising non-invasive biomarkers in diseases such as Alzheimer’s, Parkinson’s, and glioblastoma^[38-41]^. Moreover, their biocompatibility, low immunogenicity, and targeting ability position them as attractive therapeutic vehicles, capable of delivering siRNAs, anti-inflammatory agents, and chemotherapeutics across CNS barriers^[42,43]^. Stem cell-derived EVs also offer intrinsic neuroprotective and regenerative potential, with applications in neurodegenerative diseases and spinal cord repair^[44,45]^. Their ability to cross barriers such as the BBB and BRB in both directions enables them to modulate barrier integrity, immune responses, and neuroinflammatory pathways, as well as regulate neuronal signaling, glial interactions, and neuroimmune responses^[46-52]^. Their transport properties have been studied *in vitro* using BBB and BRB models and *in vivo* through rodent and zebrafish tracking studies^[53-55]^. Nevertheless, challenges persist in standardizing EV isolation, quantification, and mechanistic understanding of such transport.

Consequently, no single model can fully capture the complexity of the brain barriers. A multimodal, cross-species strategy - integrating *in vitro* platforms, rodent and zebrafish models, human stem cell-derived systems, and EV-based approaches - is essential for advancing mechanistic insights and fostering translational progress in barrier-related diagnostics and therapeutics. However, key challenges persist, including the heterogeneity of EV populations, the lack of standardized isolation protocols, and the need for scalable production methods. In this context, adherence to the MISEV^[56-59]^ guidelines is particularly relevant to brain barrier research, as it promotes methodological transparency and improves the reproducibility of EV-based experiments.

## AS IN ALL AREAS OF RESEARCH, STANDARDIZATION AND TRANSPARENCY ARE CRUCIAL TO ENSURE THE QUALITY AND REPRODUCIBILITY OF EV-BASED STUDIES ON BRAIN BARRIERS

**Figure 2:**
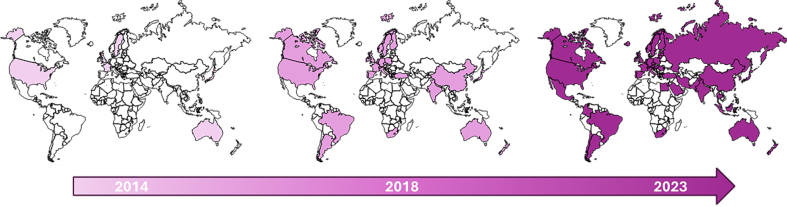
The Global Expansion of MISEV from 2014 to 2023. The growing awareness of the need for standardization, transparency, and reproducibility in EV-based research has spread worldwide like a wave. Since the first MISEV guidelines were introduced in 2014, co-authored by 15 researchers, their impact has significantly expanded. The 2018 update was endorsed by 390 authors, while the 2023 edition saw contributions from 72 principal authors and over 1,000 members of the MISEV Consortium. This map illustrates the geographic distribution of MISEV authors from its inception to its most recent update in 2023, reflecting its increasing global adoption. MISEV: Minimum information for studies of extracellular vesicles; EV: extracellular vesicle.

Given the expanding body of research on EVs, the risk of inconsistencies in nomenclature and characterization has increased, potentially undermining study transparency and reproducibility. This justified the introduction of the MISEV guidelines in 2014^[56]^, followed by updates in 2018^[58]^ and 2023^[59]^ to accommodate evolving techniques and knowledge. The widespread adoption of these guidelines, as evidenced by their global implementation [Figure 2], reflects a collective commitment among research teams to refine and standardize EV studies for improved accuracy and reproducibility. These principles are vital, particularly considering EVs’ promising applications in therapeutics. Notably, the MISEV guidelines are not restrictive but provide a flexible framework that encourages innovation and the integration of novel techniques for EV analysis and characterization. As in any field of research, EV-based studies on brain barriers benefit in robustness and reliability when conducted in accordance with MISEV recommendations, whether using *in vitro* or *in vivo* approaches.

In alignment with prior versions, MISEV2023 maintains “extracellular vesicle” as the preferred term, acknowledging the inherent diversity of EVs in terms of size, biogenesis, and function. The guidelines encourage precise classification when possible, employing descriptors such as “small EVs” (sEVs) or “medium/large EVs” (m/lEVs) based on size. A recent study by Dahlstroem *et al*. further underscores this diversity by demonstrating an alternative pathway for exosome secretion via ALG-2-interacting protein X (Alix), recruiting CD63+ late endosomes rather than following the canonical early-to-late endosome and multivesicular body (MVB) pathway^[60]^. This finding reinforces the notion that “exosome” remains a broad term encompassing heterogeneous vesicle populations.

To ensure the rigorous characterization of EVs, MISEV2023 advocates for the application of multiple orthogonal methods. These include the quantification and standardized preparation of EVs to enhance reproducibility. Researchers must distinguish EV markers to refine their characterization: (i) EV-associated transmembrane or cytosolic protein markers such as tetraspanins (CD9, CD81, CD63) and ESCRT-associated proteins; (ii) negative markers to confirm the absence of contaminants from non-EV structures, such as apolipoprotein A-1 (ApoA-1); (iii) non-EV markers that indicate specific cellular or subcellular origins such as α-actinin 4. Additionally, complementary omics approaches (proteomics, lipidomics, and transcriptomics) are recommended for in-depth EV characterization. Given the wide variety of cell types that constitute brain barrier phenotypes or contribute to their respective microenvironments, it is crucial to consider the diverse biogenesis pathways of EVs when identifying so-called “signature” proteins of specific EV populations. This consideration is particularly important for ectosomes, due to their high heterogeneity and the fact that their biogenesis can be closely linked to the cell of origin or the physiopathological context under investigation - such as in the case of protrusion-derived EVs^[61]^. For functional studies, MISEV2023 insists on stringent controls to ensure that observed effects are attributable to EVs rather than co-isolated molecules or experimental artifacts. The guidelines also underscore recent advances in EV isolation techniques, including differential ultracentrifugation, size-exclusion chromatography (SEC), and affinity-based methods. To ensure transparency and reproducibility, researchers are expected to thoroughly document methodological parameters such as processing times, centrifugation speeds, and materials used^[59]^. In the context of brain barrier research, it is further recommended to provide detailed information on the experimental models employed and, where feasible, to include validation in *in vivo* systems to support physiological relevance.

To further align with MISEV recommendations, researchers are encouraged to follow a structured checklist encompassing EV characterization, quantification, and functional validation. In *in vivo* studies, EV tracking commonly relies on labeling strategies such as CD63 tagging or staining with lipophilic dyes like PKH. However, despite their widespread use and apparent relevance for brain barrier studies^[54,62,63]^, PKH dyes present significant limitations due to their poor stability and potential for non-specific signal retention, warranting cautious application^[64]^. Additionally, MISEV2023 emphasizes the importance of data transparency and reproducibility, advocating for the use of public repositories and standardized formats - such as those supported by the EV-TRACK platform - to enhance data comparability across studies^[65,66]^. The guidelines also underscore ethical considerations, particularly in the context of in vivo experimentation and clinical translation, and highlight the need for interdisciplinary collaboration to address the technical and conceptual challenges that persist in the EV research field. While MISEV provides overarching guidelines applicable to all EV studies, it is complemented by sample-specific recommendations that are commonly used for brain barrier studies and developed by ISEV task forces: “Blood” task force provides some recommendations for studying EVs from blood and plasma referred to as MIBlood^[67,68]^; “Cerebrospinal Fluid” task force generated guidelines tailored for CSF-derived EVs^[69]^; “Conditioned Medium” task force produced a position paper with all the considerations for studying EVs from conditioned media^[70]^ (https://www.isev.org/task-forces). These specialized recommendations ensure that EV research on brain barriers accounts for sample-specific nuances, whether derived from cell culture media or biological fluids. Specifically, for biofluids such as CSF and plasma - both of which have complex and diverse compositions - strict adherence to standardized collection and pre-processing methods is essential to enhance experimental reproducibility. Filtration and/or double centrifugation steps are recommended to remove cellular debris and contaminants^[68,69]^, including platelets^[68]^. A shared recommendation across these references is to ensure higher sample quality and EV purity using SEC. Moreover, it is strongly recommended to perform a comprehensive characterization of EV-associated protein markers using complementary techniques such as nanoparticle tracking analysis (NTA), proteomics, and flow cytometry^[68-70]^. Incorporating these approaches in accordance with established guidelines significantly enhances the transparency, rigor, and reproducibility of EV research, particularly in the context of brain barrier studies.

## CONCLUSION

Expanding research on EVs is essential for deepening our understanding of their role in major physiological and pathological processes. These insights will pave the way for developing targeted therapies and identifying early disease biomarkers, ultimately improving patient care^[71]^. In the context of brain barriers, investigating EVs can enhance our ability to protect the central and peripheral nervous systems, fortify these barriers under pathological conditions, and facilitate their controlled permeation for therapeutic purposes. The prospect of using EVs to selectively target key compartments (brain, nerves, eyes) by modulating barrier integrity or influencing the fate of neural cells holds significant potential for treating associated disorders.

Adhering to MISEV and sample-specific recommendations from ISEV task forces is instrumental in ensuring the reproducibility and transparency of findings. Moreover, addressing critical open questions will propel the field forward: (i) what mechanisms govern EV transport across brain barriers?; (ii) are these mechanisms dependent on EV subtypes?; and (iii) how can these insights be leveraged to optimize therapeutic delivery to the brain? These unresolved inquiries highlight the importance of EV research in brain barrier studies, forming the foundation for future innovations in this rapidly evolving field.

## DECLARATIONS

### Copyright

© The Author(s) 2025.
